# Sleep in a mouse model of fragile X syndrome is resistant to metabolic manipulations

**DOI:** 10.1093/hmg/ddaf149

**Published:** 2025-10-02

**Authors:** Mariela Lopez Valencia, Ricardo A Velázquez Aponte, Joseph A Baur, Thomas A Jongens, Amita Sehgal

**Affiliations:** Chronobiology and Sleep Institute, Perelman School of Medicine, University of Pennsylvania, Philadelphia, PA 19104, United States; Department of Physiology and Institute for Diabetes, Obesity and Metabolism, Perelman School of Medicine, University of Pennsylvania, Philadelphia, PA 19104, United States; Department of Physiology and Institute for Diabetes, Obesity and Metabolism, Perelman School of Medicine, University of Pennsylvania, Philadelphia, PA 19104, United States; Department of Genetics, Perelman School of Medicine, University of Pennsylvania, Philadelphia, PA 19104, United States; Department of Autism Spectrum Program of Excellence, Perelman School of Medicine, University of Pennsylvania, Philadelphia, PA 19104, United States; Chronobiology and Sleep Institute, Perelman School of Medicine, University of Pennsylvania, Philadelphia, PA 19104, United States; Howard Hughes Medical Institute, Philadelphia, PA 19104, United States

**Keywords:** Fragile X Syndrome, Sleep, Metabolism, Diet

## Abstract

Fragile X Syndrome is the most prevalent known genetic cause of intellectual disability (ID), affecting around 1 in 4 000 individuals, and is also highly associated with autism spectrum disorder (ASD). Humans with the disorder and animal models display sleep and metabolic abnormalities. Given growing evidence of links between sleep and metabolism, we sought to determine if metabolic abnormalities underlie sleep deficits in mice lacking the Fragile X messenger ribonucleoprotein 1 (*FMR1*) gene. We found that metformin, a drug that targets metabolic pathways and has been shown to alleviate other symptoms in FXS, did not rescue sleep in mutant mice. Instead, metformin enhanced activity of *Fmr1* knockout (KO) mice. As a way of exaggerating possible metabolic phenotypes, we treated mice with a high fat diet (HFD) and found that although this disrupted the sleep pattern in controls, it did not impact the sleep phenotype in *Fmr1* KOs. Increased sleep during the dark phase, caused by HFD in wild type animals, was alleviated by metformin treatment. Metformin also decreased weight gain of wild type animals on a HFD, but the effect was delayed in *Fmr1* KO mice. *Fmr1* KO mice with or without metformin treatment displayed hyperphagia on a HFD, yet did not show higher weight gain than wild type. And, surprisingly, their glucose tolerance was equivalent to that of wild type mice on metformin. We suggest that *Fmr1* KO mice are better able to metabolize fat and so are relatively resistant to its negative effects on sleep and metabolism.

## Introduction

Fragile X Syndrome (FXS), which affects roughly 1 in 4000 individuals, is the most common genetic cause of Intellectual Disability (ID) and Autism spectrum disorder (ASD) [1]. FXS typically results from a repeat expansion that prevents transcription of the Fragile X messenger ribonucleoprotein 1 gene (*FMR1*) and leads to loss of the protein fragile X messenger ribonucleoprotein (FMRP), an important protein for neural development and cognitive function [1, 2]. In addition, humans and animal models with FXS show a range of sleep disturbances [3–9]. The estimated prevalence of sleep problems in individuals with FXS is high, ranging from 31%–50% [6, 7], with FXS patients often having difficulty falling asleep and increased nighttime awakenings. Sleep problems also include shorter sleep duration, lower sleep efficacy and also decreased REM sleep compared to neurotypical children [6, 8, 9].

Sleep has been increasingly linked to metabolism [10–13], and is disrupted in neurodevelopmental disorders caused by mutations in specific metabolic pathways, namely inborn errors of metabolism (IEM) [14–18]. Although FXS is not classified as an IEM, metabolic disruption has been reported. Metabolic disturbances in FXS include higher than normal BMI, and approximately 10% of people present a ‘Prader-Willi Phenotype’, which is characterized by an even higher level of obesity and a lack of satiation after meals [19–22].

Like humans, *Fmr1* KO mice display shortened sleep during the light phase and also have higher total body weight [3, 23]. Additionally, some studies suggest mitochondrial disruptions [24–26]. Thus, they are a good model for addressing whether metabolic deficits contribute to sleep phenotypes in FXS. We subjected *Fmr1* KO mice to various metabolic manipulations to assess whether they rescue or further disrupt sleep. Specifically, we asked if metformin, a first-line drug to treat type 2 diabetes, which also alleviates some symptoms in FXS as well as phenotypes in *Fmr1* KO mice and flies [27–30], rescued sleep phenotypes. To further exacerbate any underlying metabolic phenotypes, we used a 60% high-fat diet (HFD) and tested for effects on sleep in *Fmr1* KO mice. Metabolic manipulations affected sleep:wake patterns in wild type mice, which is consistent with previous findings [10], but had little effect on *Fmr1* KO mice. Indeed, sleeping patterns in *Fmr1* KO mice were remarkably resistant to metformin and HFD, and some of their metabolic parameters on HFD were better than those of wild type.

## Results

### Metformin does not rescue sleep phenotypes in *Fmr1* KO mice

**Figure 1:**
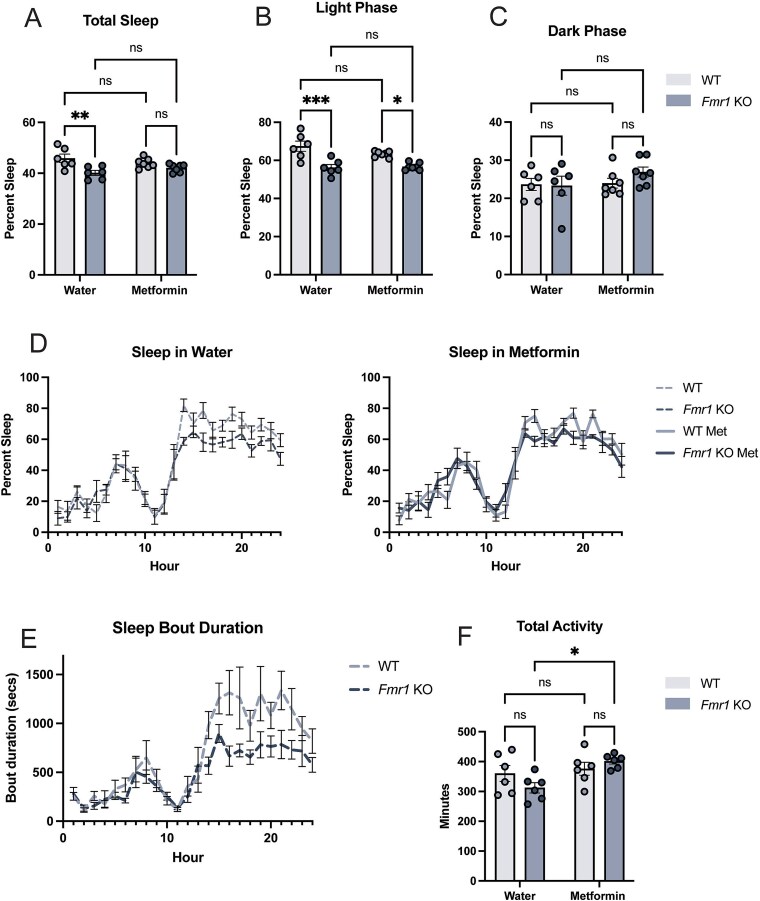
Metformin treatment does not rescue sleep in *Fmr1* KO mice, but increases their activity. (A) Average percent sleep in the 24-h dark/light cycle, (B) the light phase and (C) dark phase. (D) Sleep traces across the 24-h dark/light cycle (n = 6 for water treatment groups, n = 7 for metformin treatment groups). (E) Average bout duration in seconds every hour of the 24-h dark/light cycle (n = 11 for both genotypes). (F) Average activity over the 24-h dark/light cycle (n = 6 mice for water treatment groups, n = 6 for metformin treatment groups) data are shown as mean + − SEM ^***^P < 0.001, ^**^P < 0.01, ^*^P < 0.05, ns, not significant; calculated by a two-way ANOVA with Tukey’s multiple comparisons test.

To test for a potential therapeutic effect of metformin on sleep in FXS, we treated *Fmr1* KO mice and controls with metformin and measured their sleep patterns, hypothesizing that metformin would increase sleep in *Fmr1* KOs. Young adult mice were treated with metformin in their drinking water (2 mg/ml) for 10 days before sleep and activity were measured. We found a statistically significant difference in total baseline sleep between WT and KO mice, which was driven largely by a difference in sleep during the light phase between the genotypes, as no difference was detected during the dark phase (Fig. 1A–D). These findings are consistent with previous studies [3]. We also found that the difference in sleep amount is due to shorter sleep bout duration (Fig. 1E).

Total sleep was not significantly different between wild type and *Fmr1* KO mice following treatment with metformin, but this was due to the drug decreasing sleep in WT during the light phase rather than an increase in the sleep of *Fmr1* KO mice. Despite this effect of metformin on WT, sleep in the light phase of *Fmr1* KO mice was still significantly less than that of WT (Fig. 1B). While previous studies have been inconsistent on whether there is a change in REM sleep [4, 31, 32] we saw no difference in REM sleep in *Fmr1* KO mice compared to WT (Supplementary Fig. 1A). We also detected no difference in activity during the light phase, but activity during the dark phase was lower in *Fmr1* KO mice at baseline, aligning with previous findings [33] (Supplementary Fig. 1B and C). Metformin increased activity in *Fmr1* KO mice in both light and dark phases, resulting in an increase in total activity (Fig. 1F), with only the increase in activity during the dark phase being significant (Supplementary Fig. 1B and C). Together, these findings indicate that while metformin has small effects on WT sleep and *Fmr1* KO activity, it does not increase sleep in *Fmr1* KO mice. On the contrary, it significantly increases locomotor activity during the dark phase resulting in a total increase in activity (Supplementary Fig. 1B, F).

### A high fat diet does not exacerbate sleep phenotypes in *Fmr1* KO mice relative to WT

After testing a treatment that is known to improve metabolism, we sought to determine how sleep would be affected if we challenged *Fmr1* KO mice by feeding them a 60% high fat diet (HFD). As with normal chow, we treated a subgroup of animals with metformin to possibly alleviate the effects of the HFD. After 10 days of metformin treatment in normal chow and monitoring baseline sleep, we switched the mice to a 60% HFD. We monitored food intake for 12 weeks, after which we measured sleep and activity again. We also measured weekly weight gain up to week 16, after which we performed a glucose tolerance test.

**Figure 2:**
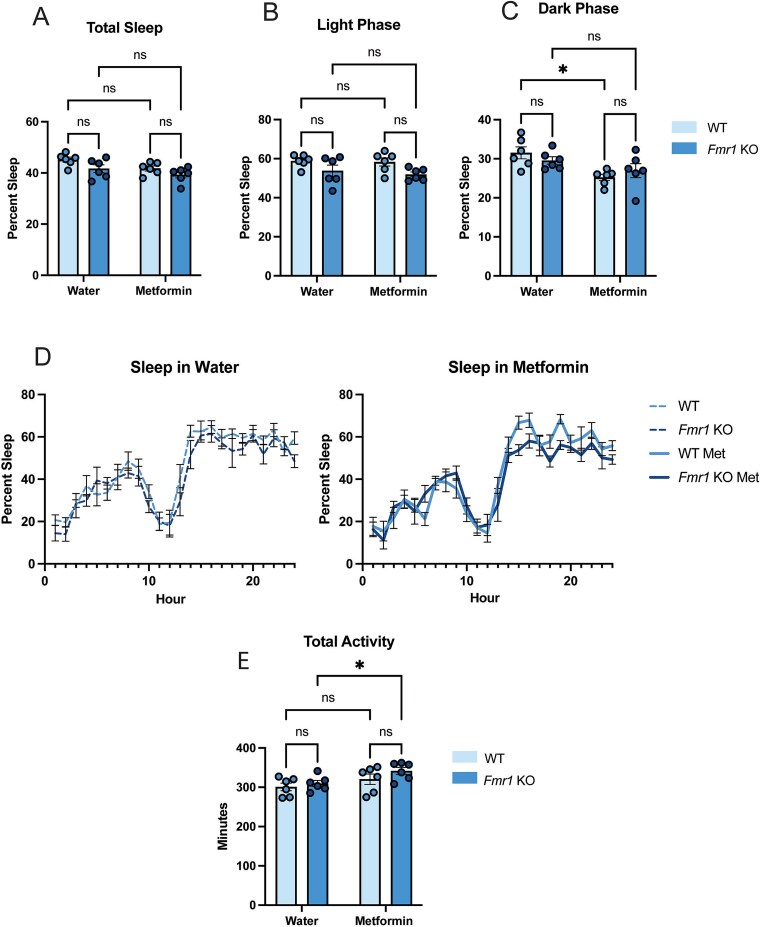
A high fat diet does not exacerbate sleep differences between WT and *Fmr1* KO mice. (A) Average percent sleep represented in the 24-h dark/light cycle, (B) the light phase, and (C) the dark phase. (D) Sleep traces across the 24-h dark/light cycle. (E) Average activity over the 24-h dark/light cycle (n = 6 for all groups). Data are shown as mean + − SEM ^*^  *P* < 0.05, ns, not significant; calculated by a two-way ANOVA with Tukey’s multiple comparisons test.

There were no differences in total sleep or sleep during the light phase between *Fmr1* KO and WT mice, but this was because HFD disrupted sleep in WT mice (Fig. 2A–D). The diurnal rhythm of sleep in WT was also dampened such that sleep decreased during the light phase and increased during the dark phase (Fig. 2A–D, Supplementary Fig. 2A and B). Thus, HFD affected sleep in WT mice, but sleep in the *Fmr1* KO mice stayed the same (Supplementary Fig. 2C and D). In WT mice, treatment with metformin normalized the sleep increase during the dark phase to that observed with normal chow (Fig. 2C, Supplementary Fig. 2A). *Fmr1* KO mice did not show less dark phase activity than controls on this diet, but, again, this is likely because of decreased dark activity (and increased sleep) in WT on a HFD (Supplementary Fig. 2E and F). Nevertheless, as with the effect on normal chow, metformin increased total activity in *Fmr1* KO mice on HFD (Fig. 2E). Neither genotype showed a difference in REM sleep on HFD (Supplementary Fig. 2G). These findings indicate that while a HFD has predictable effects on the sleep patterns of WT mice, *Fmr1* KO mice are resistant to such changes.

### High fat diet has limited effects on the metabolism of *Fmr1* KO mice despite increased food intake

**Figure 3:**
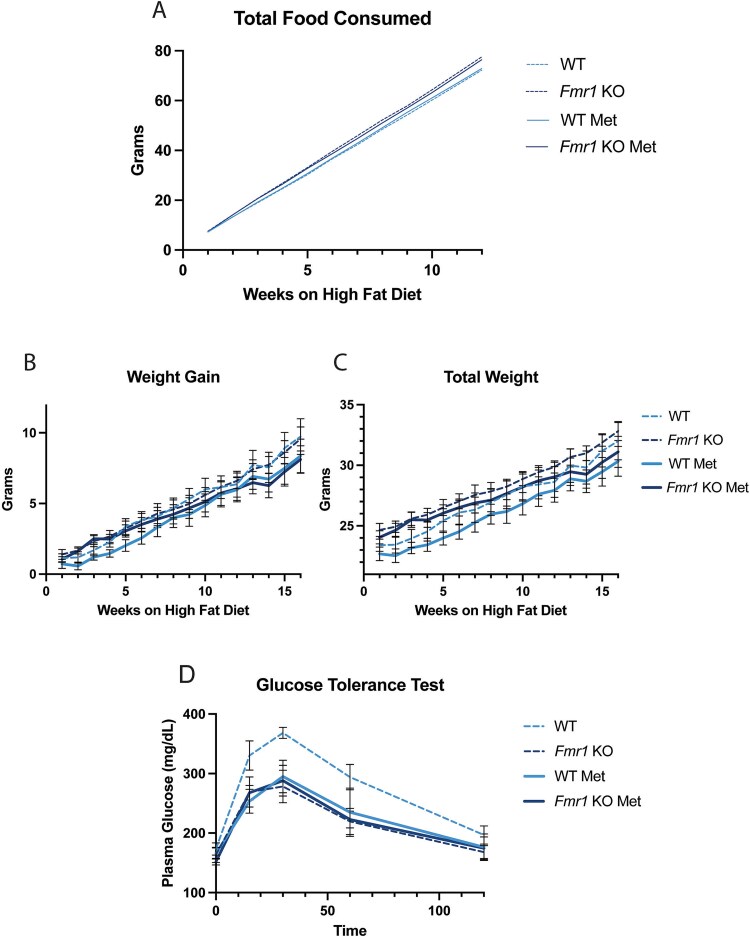
*Fmr1* KO mice show metabolic advantage over WT on a high fat diet, despite increased food intake. (A) *Fmr1* KO mice eat more than WT mice, regardless of metformin treatment, three-way anova (genotype *P* < 0.0001, treatment x genotype *P* < 0.0001, time x treatment x genotype 0.0394). (B) Metformin treatment attenuates weight gain on a HFD, ordinary three-way ANOVA (treatment *P* < 0.00001), and (C) *Fmr1* KO mice have a higher weight than WT mice, ordinary three-way ANOVA (treatment *P* < 0.0001, genotype *P* < 0.00001). (D) Glucose tolerance test, ordinary three-way ANOVA (treatment *P* = 0.0117, genotype *P* = 0.0020, treatment x genotype *P* < 0.0076) (n = 9 for all groups).

Given that sleep phenotypes were not exacerbated in *Fmr1* KO mice on a HFD, we asked how their metabolic parameters were affected by this diet relative to wild type controls. Examination of cumulative food intake over a period of 12 weeks revealed an effect of genotype such that *Fmr1* KO mice ate significantly more than the WT mice, even when treated with metformin (Fig. 3A). We also found an interaction of treatment x genotype where metformin slightly decreases feeding in *Fmr1* KO mice and slightly increases feeding in WT mice (Fig. 3A). Despite having a hyperphagia phenotype, *Fmr1* KO mice did not gain any more weight than WT (Fig. 3B). Metformin moderately reduced weight gain in both genotypes (Fig. 3B), but in *Fmr1* KO mice this was only evident after week 12, indicating that the alleviating influence of metformin is delayed in mutant mice. Consistent with this, the effect of metformin on total body weight was delayed in *Fmr1* KO mice. Mutant mice weighed more than WT in either condition, and while body weight was ultimately lower in metformin-treated animals, the effect of treatment in KO mice, again, was only evident after week 12 (Fig. 3C). WT mice trend towards a lower body weight at the start (Fig. 3C, Supplementary Fig. 3A), which might indicate that *Fmr1* KO mice are more prone to weight gain, but we find that this is not the case on a HFD; indeed, it is possible that they are better at metabolizing fats.

Finally, a glucose tolerance test showed that while WT mice on HFD take longer to clear glucose, and this is significantly rescued by treatment with metformin, *Fmr1* KO mice show the same glucose clearance with or without metformin treatment (Fig. 3D). Importantly, their glucose tolerance on HFD is better than that of wild type at baseline and equivalent to metformin-treated wild type animals. These results indicate that KO mice are relatively resistant to the effect of HFD on glucose homeostasis.

### *Fmr1* KO mice do not show differences in levels of key metabolic proteins or mitochondrial function

We asked if key metabolic proteins, in particular those implicated in metformin action such as AMPK, were changed in *Fmr1* KO mutants [34]. Given that p-AKT is an indicator of insulin sensitivity [35] and a target of metformin, we looked at the expression of p-AKT and p-AMPK. We focused on the liver, as this is a metabolically relevant tissue, and the hippocampus since it is implicated in learning and memory, but found no differences between wild type mice and *Fmr1* mutants (Supplementary Fig. 4A and B). Several studies have reported mitochondrial deficits with loss of *Fmr1*, for example one study showed decreases in Drp-1 and Mitofusin-2 in *Fmr1* KO cells, another showed differences in PGC1-⍺ in *Fmr1* KO cells and *dfmr1* mutant flies [24–26, 36, 37]. Because of this, we measured Drp-1, Mitofusin-2, PGC-1⍺, and Sirtuin-1 as additional markers of mitochondrial health, yet we were unable to find differences in any of these proteins in the hippocampus of our *Fmr1* KO mice (Supplementary Fig. 5A–D). As a proxy for mitochondrial mass and health, we also quantified the levels of cytochrome-b in the cortex through qPCR (Supplementary Fig. 5E). While the level of cytochrome-b trended lower, the difference was not statistically significant.

**Figure 4:**
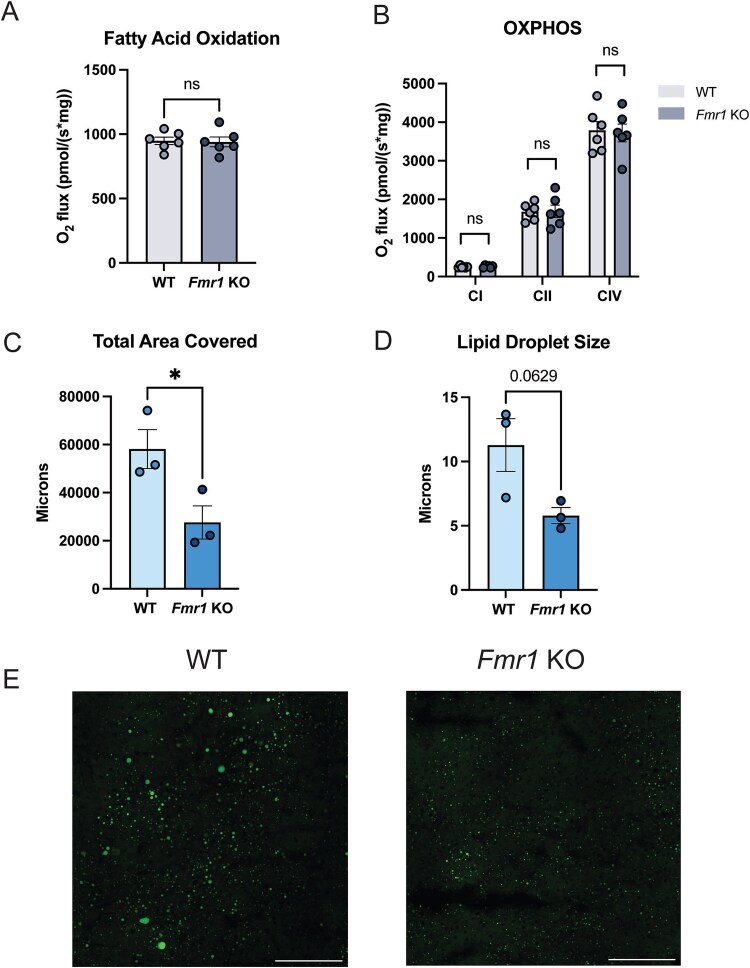
*Fmr1* KO mice show no difference in mitochondrial function at baseline but have lower lipid droplet content in the liver on a HFD. (A) Fatty acid oxidation rate and (B) mitochondrial complex function are not different among genotypes (n = 6 for both groups). (C) Lipid droplet accumulation is higher and (D) lipid droplet size trends lower in WT mice compared to *Fmr1* KO mice, scale bars represent 140 microns. (E) BODIPY staining in livers (n = 3 for moth groups). Data are shown as mean + − SEM ^***^  *P* < 0.001, ^**^  *P* < 0.01, ^*^  *P* < 0.05, ns, not significant; calculated by unpaired t-test.

Since the cytochrome-b levels trended lower we decided to directly assay mitochondrial function. We measured fatty acid oxidation and mitochondrial complex activity in the livers of both genotypes on normal chow. We saw no differences in fatty acid oxidation or complex I-, II-, and IV-dependent respiration (Fig. 4A and B). To address the basis of the HFD phenotype, where *Fmr1* KO mice did not gain any more weight despite eating more, we asked if they developed fatty livers typically induced by such diets [38]. Staining livers for lipid droplets (LDs) revealed higher accumulation of LDs, as well as a trend towards higher LD size, in WT mice relative to mutants (Fig. 4C–E). This further demonstrates that *Fmr1* KO mice are resistant to some of the negative metabolic effects of a HFD.

## Discussion

We studied the relationship between sleep and metabolism in a mouse model of FXS by manipulating metabolism in different ways and determining how these manipulations affect sleep. Studies in the fruit fly and mice have shown metabolic abnormalities, some of which have parallels in people with Fragile X Syndrome [23, 37, 39–41]. In fruit flies, an increase in insulin expression was seen, but levels in the circulating hemolymph have not been measured [30]. In *Fmr1* KO mice and FXS patients, a decrease in serum insulin levels is reported [39, 41].

While the effects on insulin in flies and mammals seemingly differ, both fly and mouse models of FXS display increased PI3K signaling that is linked to behavioral and cognitive phenotypes [30, 42]. Both models also display decreased lipid and carbohydrate stores [39, 42]. *Fmr1* KO mice appear to have high insulin sensitivity, as well as lower amounts of blood glucose and fat [23, 39], which is surprising given that some phenotypes are rescued by metformin, the first-line drug to treat Type 2 Diabetes because of its ability to decrease blood glucose. Metformin was shown to rescue behavioral grooming, aspects of the ERK pathway and dendritic abnormalities in *Fmr1* KO mice [29]. In addition, while sleep was not investigated in this context, metformin rescued olfactory and courtship-based memory in the fly model of FXS [30]. Metformin also had positive effects on people with Fragile X, alleviating symptoms such as hyperactivity, irritability and social responsiveness, among others [27, 28].

People with FXS show sleep disruption, and studies in mice have shown a decrease in sleep during the light phase as well as a difference in circadian rhythmicity [3, 4, 7–9, 43]. We replicated the effect on sleep using a piezo monitoring system. While this system has not been used previously for *Fmr1* KO mice, it is now used widely for sleep research. Importantly, details of the sleep phenotype with the piezo mirrored those observed by beam break and video recording assays [3, 5]. In our efforts to identify a mechanism linking metabolism and sleep, we determined whether metabolic pathways implicated in circadian rhythms/sleep, and also targets of metformin, are altered in mutant mice. Notably, the pathways we examined, p-AKT and p-AMPK signaling, are also affected by a HFD [35, 44]. While there is a disagreement on whether the loss of FMRP affects levels of p-AKT/AKT in the cortex [45, 46], differences have been observed in the hippocampus and hippocampal neurons [46, 47], which we were unable to detect in our study. We also saw no difference in levels of p-AKT in the liver or differences in p-AMPK in the hippocampus, which aligns with previous findings [29, 39]. We speculate that the activity of these metabolic pathways may change in specific tissues of knockout mice depending on endogenous or environmental factors such as diet, age and housing conditions.

Based on work linking loss of FMRP to deficits in proteins related to mitochondrial fission and fusion, such as Drp-1, Mitofusin-2, and PGC-1⍺ [25, 36, 37], we measured these in our mice, but did not detect any differences between *Fmr1* KO and WT mice. It is worth mentioning that in the mouse studies, differences in PGC1-⍺ were found specifically in synapses, and the differences in Drp-1, Mitofusin-2 were found in cultured neurons, while we measured the whole hippocampus. It is possible that the intact animal compensates for cellular deficits through cell–cell communications or circuit activity. We also measured the levels of cytochrome-b in the cortex as a proxy for mitochondrial mass and found that it trended lower in *Fmr1* KO mice cortex (*P* = 0.0689) which is consistent with the decreased levels of cytochrome related mRNA levels seen previously [25]. We acknowledge that there are other ways to measure mitochondrial mass and function, but together with the lack of an effect on DRP-1 or PGC1-⍺, our data do not reveal obvious mitochondrial deficits in brains of *Fmr1* KO mice. Discrepancies between studies could also arise from different rearing and handling conditions. For instance, our mice were not littermates and were single-housed for purposes of accurate monitoring of sleep and food intake; they were also not the same age as the mice previously studied, all factors that can contribute to the variability in our results compared to past studies. Interestingly, reported changes in the levels of PGC1-⍺ in young *Fmr1* KO mice go in similar directions as observed in the fly model, but in mature mice they are affected in the opposite direction, so this regulation by FMRP is likely to be complex [25, 37]. Finally, we measured beta oxidation as well as complex-dependent mitochondrial function in the liver and found no difference.

Metformin effectively rescues several phenotypes, including behaviors in flies and mouse models of FXS, but we did not find rescue of the sleep deficit in *Fmr1* KO mice. With metformin treatment, WT mice show a slight reduction in sleep, but *Fmr1* KO mice stayed the same, with the exception of an increase in activity. Some studies have also found a hyperactivity phenotype at baseline in the *Fmr1* KO mice, but is normally only seen with specific methods like the open field arena [23, 33, 39]. Differences in REM sleep between *Fmr1* KO and WT mice are controversial [4, 32] and were not evident in our study.

People with FXS usually have a higher BMI than the general population, and mouse studies have also shown increased body weight in *Fmr1* KO mice [19, 23]. We asked if we could exacerbate this increase in body weight and potentially other metabolic phenotypes, which might further disrupt sleep in the *Fmr1* KO mice. A 60% HFD did not increase body weight gain more so in *Fmr1* KO mice than in WT. Although food intake was higher in *Fmr1* KO over the course of the 12 weeks of HFD, mutant mice did not gain any more weight than WT, and the weight-lowering effects of metformin were only effective in *Fmr1* KO mice after 12 weeks of treatment. In addition, examination of liver lipid droplets revealed less fat in the livers of *Fmr1* KO mice, indicating that mutant mice metabolize fat better. A glucose tolerance test of animals fed a HFD showed that *Fmr1* KO mice clear glucose faster than WT, equivalent to the clearance seen in WT mice treated with metformin. Treatment with metformin did not further accelerate glucose clearance, suggesting that even metabolic parameters in *Fmr1* KO mice may be relatively resistant to this drug.

Overall, our data support the idea that the *Fmr1* KO body composition consists of more lean mass, and that the knockouts are better at lipolysis and lipid use for energy [23, 39]. Interestingly, people with FXS also show reduced cholesterol as well as reduced low-density lipoprotein and high-density lipoprotein levels, despite their higher BMI [48]. The fact that KO mice clear glucose more rapidly even on a HFD suggests that the altered body composition and metabolic advantages of *Fmr1* KO remain on a fat-based diet. HFD disturbed the sleep patterns in WT mice by decreasing the sleep during the light phase and increasing the sleep during the dark phase, with these effects partially normalized by metformin administration. While other studies of WT mice on HFD have reported an increase in sleep during the light phase [10, 11], our results are consistent with human studies where people with obesity have low sleep quality at night and experience sleepiness during the day [12]. Additionally, metformin treatment in humans leads to better sleep quality, much like we see with metformin decreasing the nighttime sleep increase caused by HFD [13, 49]. Surprisingly, these changes were not seen in *Fmr1* KO mice, just as we did not see major changes in metabolic measurements. We speculate that the metabolic makeup and body composition of *Fmr1* KO mice protects not only against the metabolic deficits induced by a HFD, but also against the sleep abnormalities it normally causes.

Our goal for this study was to investigate metabolic deficits as an underlying cause of the sleep deficit in *Fmr1* KO mice. However, we were unable to find any manipulation that changed sleep in the *Fmr1* KO mice. We note that other drugs such as rapamycin, an TORC1 pathway inhibitor, and Zatolmilast, a cyclic AMP pathway inhibitor, are also unable to rescue the sleep deficit in the mice [45, 50]. It appears as though the sleep deficit in *Fmr1* KO mice is very robust and is either non-metabolic in origin or caused by multiple metabolic deficits, which are not rescued by any single drug.

## Materials and methods

### Mouse breeding

WT female mice (C57BL/6 J) and *Fmr1* KO (B6.129P2-Fmr1*^tm1Cgr^*/J) mice were obtained from Jackson Laboratories and bred to obtain *Fmr1* KO heterozygous females. We then bred the heterozygous females with WT and *Fmr1* KO male mice to obtain *Fmr1* KO and WT mice. All live animal experiments were performed according to protocols approved by the Institutional Animal Care and Use Committee of the University of Pennsylvania (protocol #806387) and in accordance with the guidelines set by the National Institute of Health.

### Metformin administration

Male WT and *Fmr1* KO mice in a C57BL/6 background were single-housed once they were 7–8 weeks of age. Mice were treated with 2 mg/ml metformin (Sigma, cat #D150959) in their water (about 350 mg/kg/day), which is a dose used in other mouse models with neurological deficits and one that has been shown to be anti-hyperglycemic in diabetic models [51–53]. Metformin treatment was given for 10 days before the initiation of sleep monitoring and the treatment was continued until the mice were sacrificed. The water was changed weekly, and mice were provided food *ad libitum* during the entirety of metformin treatment. For the sleep monitoring system, animals were single-housed and tissue was collected a week after the 7 days of sleep measurement were done, mice were single housed until sacrificed. For the long-term metformin treatment along with HFD, the metformin doses stayed the same, and mice stayed singly housed.

### Sleep monitoring system

Sleep was monitored using a non-invasive piezoelectric system (Signal Solution, Lexington, KY, USA). This method has been validated with electroencephalogram (EEG) [54]. The system consists of a cage with an open bottom that allows a direct contact of the animal and the sensor on the floor of the cage. The sensors generate a pressure signal. Output signals were amplified and filtered between 0.5 and 10 Hz. The amplified signals were analogue-to-digital (A/D) converted at a sampling rate of 128 Hz using the LabView 7.1 software (National Instruments, Austin, TX, USA). The signals were analyzed over tapered 8-s windows at a 2-s increment and a decision statistic was computed and classified using a linear discriminate classifier. Data were binned at each hour on a rolling average percentage sleep during both light and dark phases. For the sleep trace across the 24- h day the data represents percent sleep the mice had during each hour. For sleep fragmentation, data were binned by length of individual bouts to calculate hourly mean bout length across the 24-h day (in seconds).

### Activity

Activity was monitored with the same system as the sleep. In this system activity is not computed when the animal moves, but instead it is counted as activity when the activity measured computed in SleepStats is proportional to the rate and relative force of motions over short-time intervals (4 s). Total activity was computed by taking activity levels every 10 minutes and adding them over the 12-h light and dark cycle.

### High fat diet feeding, food intake and weight gain

60% HFD was administered following the initial 10 days of metformin treatment and after the baseline sleep and activity were established. This diet, along with the metformin, was administered for 20 weeks until the mice were sacrificed. Food was weighed every other day for 12 weeks, and mice were weighed every week for 16 weeks. For accurate measurement of food intake for each individual mouse and to avoid excessive disintegration of food, the mice stayed single housed.

### Glucose tolerance test

Mice were fasted for 5 h prior to glucose injection. Glucose was injected intraperitoneally at 0.5 g/kg. Samples of blood were taken by tail venesection at 0, 15, 30, 60, and 120 mins. Glucose was measured in freely moving unrestrained animals using a glucometer. Mice were only given water *ad libitum* throughout the duration of the test.

### Immunoblotting

Immunoblotting was used to detect expression of Akt (Cell Signaling #9272,), p-Akt (S473) (Cell Signaling #4060S), AMPK (Life Technologies #MA5–15815) P-AMPK (Cell Signaling #2535), Drp-1 (Cell Signaling #8570S), p-Drp1 (S637) (Cell Signaling #6319), Mitofusin-2 (Cell Signaling, #9482), Sirtuin 1 (Cell Signaling #9475), PGC-1⍺ (sigma-Aldrich #ST1202-1SET) and β-Tubulin (Cell Signaling #15115). In brief, hippocampus and liver protein (20ug) was extracted using RIPA buffer with protease and phosphatase inhibitors. Extracted protein was analyzed by electrophoresis on SDS-PAGE gels and transblotted on nitrocellulose membrane (0.45uM). Membranes were blocked with blocking buffer and incubated with relevant primary antibody at a 1:1000 dilution. After washing, bound antibody was detected using anti-rabbit/anti-mouse at a 1:5000 dilution linked to HRP.

### Q-PCR

RNA was extracted using RNeasy mini kit (Qiagen) and reverse transcribed to cDNA using random hexamers. Real-time polymerase chain reaction (PCR) was performed using Sybr Green PCR Master Mix (Applied Biosystems). Assays were run on ViiA7 Real-Time PCR system (Applied Biosystems). Relative gene expression of cytochrome b (forward: TATTCCTTCATGTCGGACGA; reverse: AAATGCTGTGGCTATGACTG; probe: ACCTGAAACATTGGAGTACTTCTACTG) was calculated using the ΔΔCt method normalizing to Rplpo (forward: AGATTCGGGATATGCTGTTGGC, reverse: TCGGGTCCTAGACCAGTGTTC).

### Mitochondrial isolation

Mitochondrial isolation was performed as described previously [55]. Briefly, ~ 100 mg of liver tissue was finely minced and homogenized in 1.5 ml of ice-cold mitochondrial isolation buffer (MIB; 210 mM mannitol, 70 mM sucrose, 1 mM EDTA, and 10 mM HEPES; pH adjusted to 7.2) supplemented with 0.25% (w/v) fatty acid–free bovine serum albumin (BSA, Roche). Homogenization was performed for 12 strokes using a Potter-Elvehjem tissue grinder set at 500 RPMs in ice. Mitochondria were then isolated by differential centrifugation. The homogenate was first centrifuged at 1000 × g for 10 minutes at 4°C. The supernatant was then collected and centrifuged for 10 minutes at 10000 × g at 4°C. The mitochondrial pellet was washed in MIB without BSA for 10 minutes at 10000 × g at 4°, resuspended in MIB without BSA, and kept on ice. Protein concentration was then determined by the Pierce bicinchoninic acid assay (Thermo Fisher Scientific, 23 225).

### High resolution Respirometry

Mitochondrial respiration rates were assessed using an Oroboros Oxygraph-2 k (Oroboros Instruments, Austria). Briefly, ~ 200 μg of isolated liver mitochondria were added to the respiration chamber containing pre-warmed MiR05 respiration buffer (0.5 mM EGTA, 20 mM taurine, 2 mM MgCl_2_, 110 mM sucrose, 60 mM K-lactobionate, 10 mM KH_2_PO_4_, 10 mM K-HEPES, and 1 mg/mL BSA, pH adjusted to 7.1). For OXPHOS complexes, 20 mM pyruvate and 10 mM malate were added and allowed to stabilize for approximately 10 minutes. State-3 respiration was achieved after the addition of ADP to a final concentration of 1 mM. Complex I-dependent respiration was then blocked by adding 0.5 μM Percidin A (Cayman Chemical, 15 379). Succinate at 20 mM was then added to stimulate Complex II-linked respiration, followed by the addition of 5 μM Antimycin A (Sigma, A8674). Complex IV-linked respiration was assessed by the addition of a mixture of 0.5 mM N, N, N′, N′- tetramethyl-p-phenylenediamine (TMPD) and 2 mM ascorbate, allowing the reading to stabilize, and followed by the addition of 5 mM NaN_3_. Quantification of Complex IV-linked respiration was calculated as the difference between the TMPD/ascorbate reading and the NaN3 reading. For assessing fatty acid oxidation, 10 mM malate was added and allowed to stabilize for approximately 10 minutes. Palmitoylcarnitine at 0.025 mM (Sigma, P4509) was then added, followed by 1 mM ADP. All runs were normalized to protein concentration. Data were analyzed using the DatLab software 4.3 (Oroboros Instruments, Austria).

### Tissue sectioning and staining

Mice were cervically dislocated, liver samples were immediately collected and flash frozen in liquid nitrogen. The tissue was then transferred to Optimal Cutting Temperature (OCT) compound (Tissue Tek) and stored in -80C. Liver was sliced in sections of 20um on Leica CM3050 cryostat, dry mounted on Superfrost Plus slides and stained using BODIPY 493/503 (Fisher-D3922). Slices were washed in PBS 2 × 10 minutes after which BODIPY was added for 1 h at a concentration of 1:1000, then washed again 3 × 5 minutes. Stained slices were then sealed using Vectashield Antifade Mounting Medium (Vector Laboratories H 100–10) and a coverslip.

## Supplementary Material

SupplementaryFigure_Legends_ddaf149

SupplementaryFigure1_ddaf149

SupplementaryFigure2_ddaf149

SupplementaryFigure3_ddaf149

SupplementaryFigure4_ddaf149

SupplementaryFigure5
