# Supplementary material for: Sleep in a mouse model of fragile X syndrome is resistant to metabolic manipulations
Source: Hum Mol Genet. 2025 Oct 2;34(22):1874–83. doi: 10.1093/hmg/ddaf149 (PMC12581825; doi:10.1093/hmg/ddaf149)
Supplement: SupplementaryFigure_Legends_ddaf149 [file supplementaryfigure_legends_ddaf149.docx]

Supplementary Figure 1. No differences in REM sleep at baseline and increased activity in *Fmr1* KO mice when treated with metformin. A) Percent REM in water and metformin B) activity during the dark phase and C) activity during the light phase (n = 6 mice for all groups). Data are shown as mean +- SEM ** *P* < 0.01, * *P* < 0.05, ns, not significant; calculated by a two-way ANOVA with Tukey’s multiple comparisons test

Supplementary Figure 2. High Fat Diet disrupts sleep in WT mice but is unaffected in *Fmr1* KO mice. A) Percent sleep in WT on normal chow compared to HFD in the dark phase and the B) light phase, C) percent sleep in *Fmr1* KO mice on normal chow compared to HFD in the dark phase and the D) light phase (n = 6 mice for water treatment groups, n = 7 for metformin treatment group in normal chow). E) Activity in the dark and F) light phase. G) There is no difference in REM sleep. Data are shown as mean +- SEM *** *P* < 0.001, ** *P* < 0.01, ns, not significant; calculated by a two-way ANOVA with Tukey’s multiple comparisons test

Supplementary Figure 3. Initial body weight trends higher on *Fmr1* KO mice. A) Initial body weight of WT and *Fmr1* KO mice (n = 9 for both genotypes). Calculated by unpaired t-test with Welch’s correction.

Supplementary Figure 4. There is no difference in p-AKT or p-AMPK levels between WT and *Fmr1* KO mice. A) p-AMPK and p-AKT levels in the hippocampus (treatment effect in p-AMPK in the hippocampus *P* = 0.0453) (n = 5 for both groups) and B) the liver (n = 3 both groups). Calculated by a two-way ANOVA with Tukey’s multiple comparisons test.

Supplementary Figure 5. Representative western blots of mitochondrial markers in *Fmr1* KO mice compared to WT, A) Sirtuin-1, B) PGC1-⍺, C) Mitofusin-2, D) Drp-1, and E) and Cytochrome b mRNA levels in the cortex. (n = 5 for all groups). Calculated by unpaired t-test with Welch’s correction.
