# Supplementary figures and images for: Sleep in a mouse model of fragile X syndrome is resistant to metabolic manipulations
Source: Hum Mol Genet. 2025 Oct 2;34(22):1874–83. doi: 10.1093/hmg/ddaf149 (PMC12581825; doi:10.1093/hmg/ddaf149)

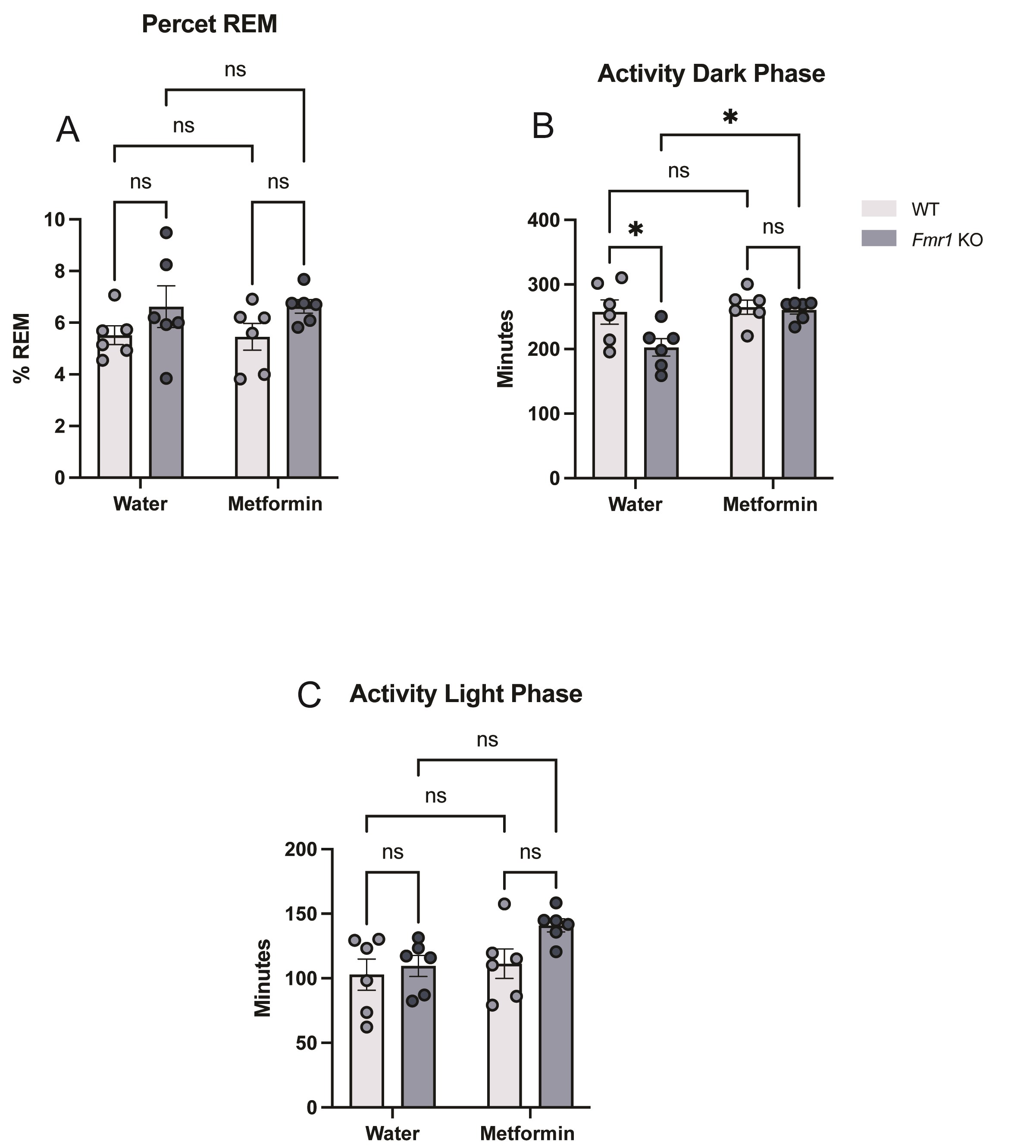

Supplement: SupplementaryFigure1_ddaf149 [file supplementaryfigure1_ddaf149.jpeg]

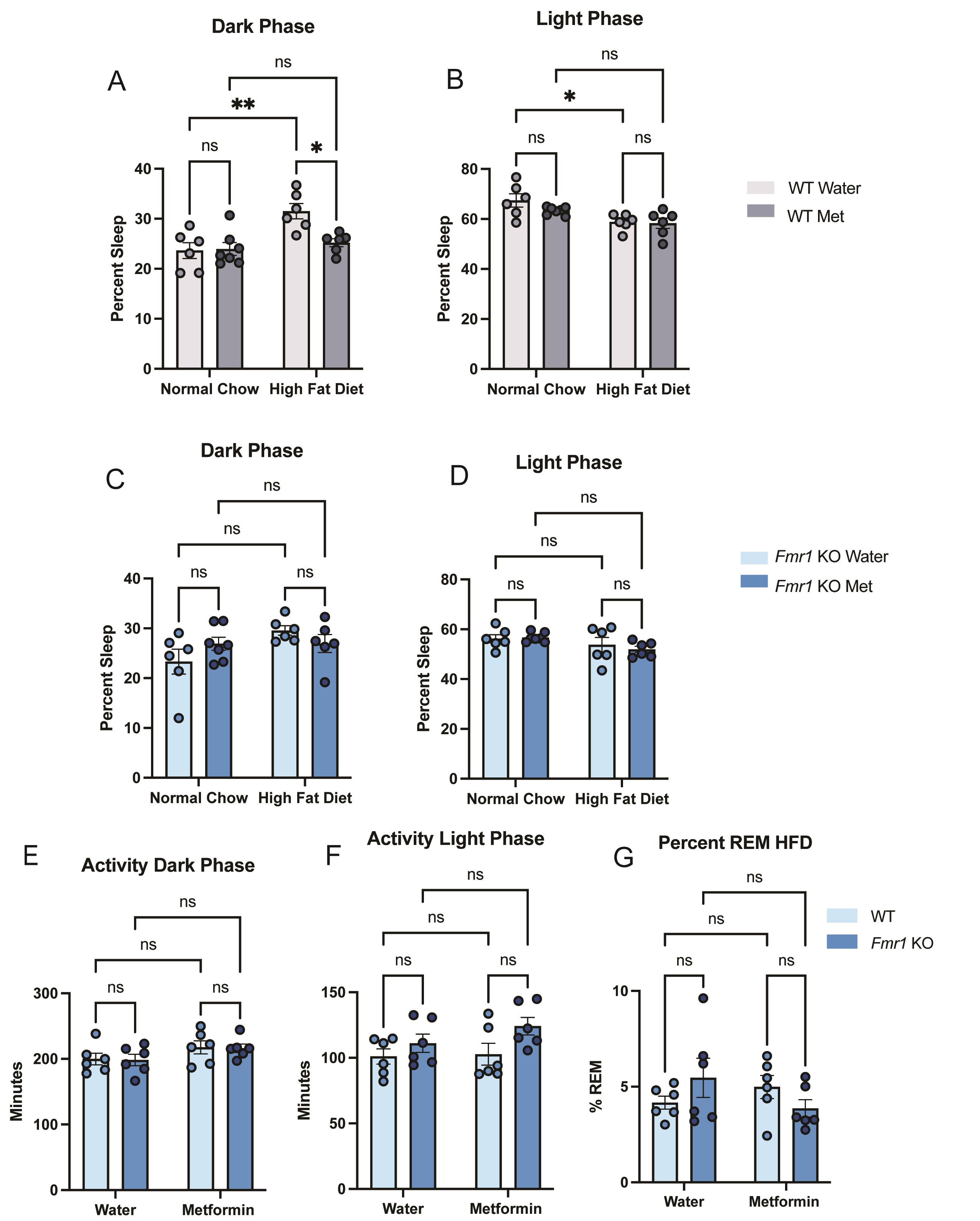

Supplement: SupplementaryFigure2_ddaf149 [file supplementaryfigure2_ddaf149.jpeg]

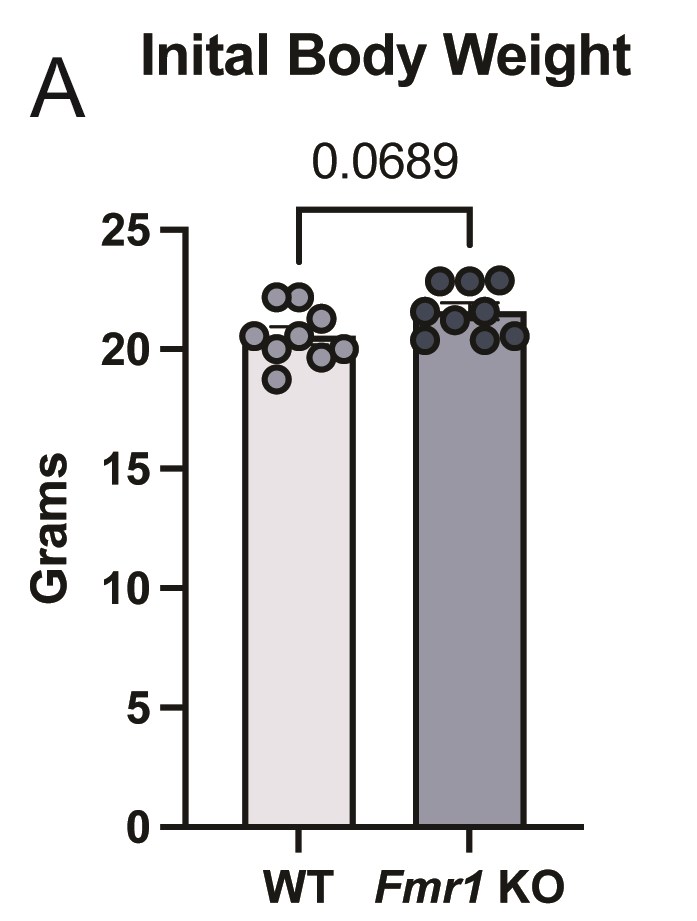

Supplement: SupplementaryFigure3_ddaf149 [file supplementaryfigure3_ddaf149.jpeg]

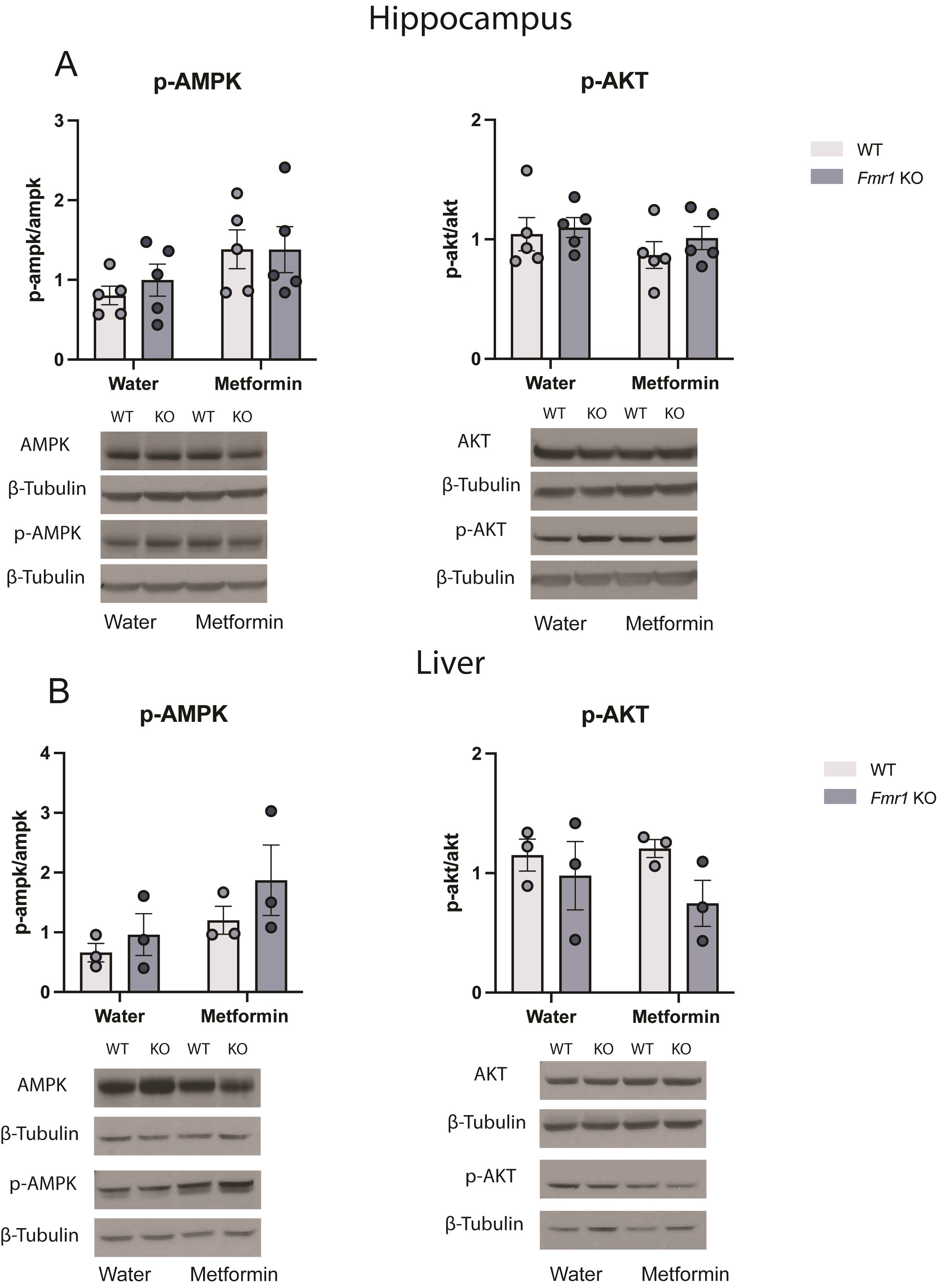

Supplement: SupplementaryFigure4_ddaf149 [file supplementaryfigure4_ddaf149.jpeg]

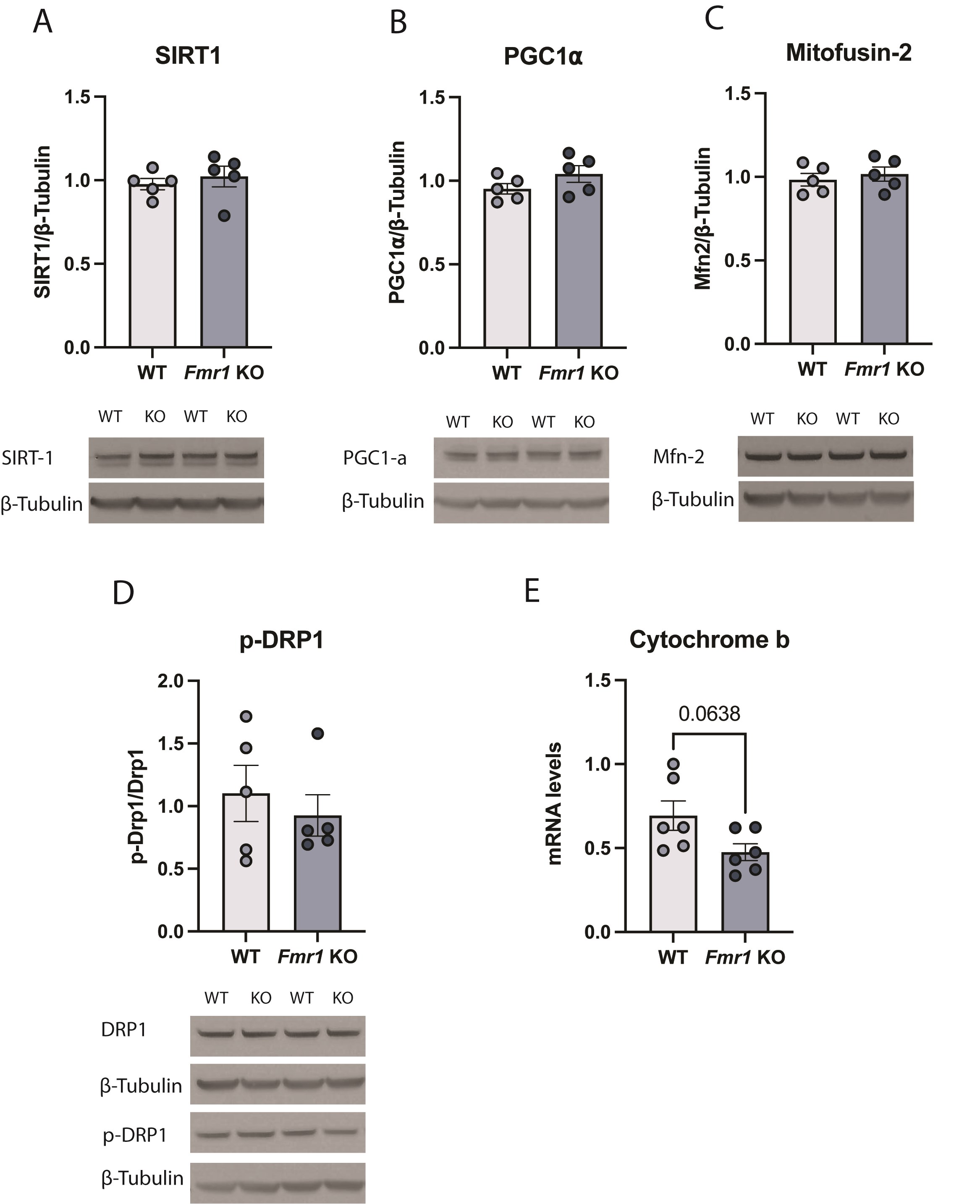

Supplement: SupplementaryFigure5 [file supplementaryfigure5.jpeg]
